# Preservation of minimally processed carrots using the combination of ultrasound and mild heat ascorbic acid

**DOI:** 10.1016/j.ultsonch.2024.107086

**Published:** 2024-09-27

**Authors:** Jiayi Wang, Ning Zhou, Sen Ma, Xiaofei Yang, Jun Xing

**Affiliations:** a National Demonstration Center for Experimental Biology Education, Xinjiang Key Laboratory of Biological Resources and Genetic Engineering, College of Life Science & Technology, Xinjiang University, Urumqi 830046, China; b College of Grain Science and Technology, Shenyang Normal University, Shenyang 110034, China

**Keywords:** Ultrasound, Minimal processing, Pathogens, Disinfection

## Abstract

Ultrasound (US) in combination with chemical disinfectants is an efficient and cost-effective hurdle technology for disinfecting minimally processed produce (MPP). However, the demand for non-chemical disinfection methods is increasing. In addition, chemical methods have been ineffective in simultaneously improving the physiological properties and inactivating pathogens in MPP. In this study, a novel and safe method called mild heat ascorbic acid (MHAsA; 1 % AsA at 50 °C) was combined with US to process minimally processed carrots. Physiological properties and microbial inactivation efficacy were analyzed during the storage period (0–5 days). The findings indicated that US-MHAsA induced the highest levels of antioxidant enzymes (superoxide dismutase and catalase) activities and accelerated the glutathione-ascorbate cycle, resulting in lower reactive oxygen species (ROS) and malondialdehyde content compared to US and MHAsA. The efficacy of US-MHAsA in inactivating phenylalanine lyase, the initial enzyme in the lignin synthesis process, was lower than that of US. On the other hand, its ability to inactivate cinnamyl alcohol dehydrogenase, the final enzyme in the process, was better than that of both US and MHAsA. However, there were no significant differences in lignin content among the three groups. The inactivation efficacy against enzymes (polyphenol oxidase and peroxidase) involved in browning was consistent across the three treatments. Analysis of the disinfection efficacy against *Escherichia coli* O157:H7 and *Salmonella* Typhimurium revealed that US-MHAsA achieved the lowest cross-contamination incidence (10–12 %) during washing, which was significantly lower than the incidence achieved by US (75–82 %). During the period from day 0 to day 5, two pathogens on carrots in the control group increased from 6.25 to 6.64 log CFU/g, while the lowest counts were observed in the US-MHAsA group, decreasing from 4.44 to 3.74 log CFU/g. However, the counts in the US group increased from 5.22 to 6.32 log CFU/g, and the counts at day 5 were not significantly lower than the control. These findings indicate that US-MHAsA is a novel hurdle technology that effectively reduces the risk of pathogen contamination and enhances the ability of MPP to scavenge ROS.

## 1 Introduction

Fresh-cut fruits and vegetables, also known as minimally processed produce, are increasingly popular among consumers due to their convenience. These washed, cut, and packaged fruits and vegetables are used as ready-to-cook products and are widely available in restaurants, supermarkets, and fast food establishments. These products can significantly reduce cooking time and minimize food waste [1]. Fresh produce may deteriorate in quality after being cut, including color, flavor, and nutrients loss. Furthermore, surface microorganisms can readily transfer to the cut surface and become internalized, resulting in contamination and subsequently leading to produce spoilage [2]. To address these challenges, appropriate preservation methods must be implemented to ensure the freshness and safety of fresh-cut produce.

Traditionally, preservation methods were employed either for quality enhancement or for microbial inactivation, and it was rare to satisfy both objectives simultaneously. Hurdle technology combines several preservation methods to create an enhanced effect, simultaneously preserving the safety, stability, nutritional value, and taste of fresh produce [3]. Non-residual physical techniques have emerged as a focal point in fresh-cut produce processing research. Among the physical methods, ultrasound (US) preservation is a low-cost and user-friendly technology that can remove dirt and microbes from the surface of fruits and vegetables, as well as inactivate enzymes [4]. Oda et al. [5] utilized US to wash spinach and maintain its freshness. Sun et al. [6] found that US can effectively delay the browning of sliced mushrooms during storage. US was generally combined with chemical agents to form hurdle. For instance, Hou et al. [7] combined US with NaClO to wash fresh-cut cucumber and found that the effectiveness of US-NaClO against *Listeria monocytogenes* was higher than single treatment.

With the increasing focus on economical and chemical-free preservation methods, it is essential to identify a non-chemical approach that can be combined with US. Among other physical techniques, thermal processing remains unrivaled in terms of cost-effectiveness. Mild heat (MH) treatment has been found to have good preservation effects without affecting sensory quality [8]. This method entails washing fruits and vegetables with water at temperatures ranging from 35 to 60 °C for periods of 10 s to 1 h [8]. This method differs from long-term high-temperature disinfection treatments and is a more gentle method to enhance the ability of fruits and vegetables to scavenge free radicals and maintain cell membrane integrity and function. The browning of sliced mushrooms was inhibited by MH (40°C, 5 min) by increasing antioxidant enzyme activity and inactivating browning-related enzymes [9]. Siddip et al. [10] treated fresh-cut onion slices with hot water at temperatures of 50, 60, and 70 °C for 1 min and observed higher antioxidant activity and color value compared to the control. The effectiveness of US-heat treatment in delaying phenolic compounds accumulation, inducing antioxidant activity, and inducing phenylalanine lyase (PAL) activity in fresh-cut lotus root, was significantly higher than single treatment [11].

Natural product preservation refers to the utilization of antioxidants or antimicrobial agents that are naturally present in plants. While natural products offer the benefit of low-toxicity, they are typically expensive. However, ascorbic acid (AsA), an essential natural antioxidant present in fruits and vegetables, is extensively used as a low-cost food additive in food industry. AsA has been utilized to maintain the quality properties of fresh-cut produce. Zhao et al. [12] examined the mechanism of AsA in inhibiting yellowing in fresh-cut yam and discovered that AsA can effectively reduce energy metabolism and inhibit pigment biosynthesis. Zhou et al. [13] studied the effect of AsA on the wound healing response of fresh-cut potato slices and found that browning, weight loss, and respiratory rate were significantly delayed. Only recently, we used mild heat ascorbic acid (1 % solution at 50 °C; MHAsA) to wash fresh-cut carrot and observed the increased effectiveness in inactivating pathogens, inducing antioxidant capacity, and delaying lignification, as compared to AsA and MH [8]. However, it is unknown whether the effectiveness was further increased after combining MHAsA with US. Carrots exhibit a distinct post-harvest physiological response compared to other produce, undergoing lignification upon cutting, which results in a white appearance. Furthermore, carrots are susceptible to microbial contamination, a characteristic shared with other produce. Therefore, this study aims to determine the effectiveness of US-MHAsA in inactivating food-borne pathogens (*Escherichia coli* O157:H7 and *Salmonella* Typhimurium) and inducing physiological metabolism. Fresh-cut carrot was selected as the model for analysis.

## 2 Materials and methods

### 2.1 Sample preparation

Carrots with no mechanical damage, rot, or deterioration were purchased from the local market on the day of the experiment and cut into cubes (8 mm × 8 mm × 8 mm) using a dicer (MDQ20; MUPOOL, Foshan, China) [14]. The resulting sample was then dewatered using a manual salad spinner that had been sterilized with 75 % ethanol.

### 2.2 Inoculation

*E. coli* O157:H7 (ATCC700728) and *S*. *Typhimurium* (ATCC14028), which have previously been reported for use in fresh-cut produce inoculation [8], [15], [16], were employed in this study. A single colony of each pathogen was transferred to nutrient broth (Hopebio, Qingdao, China) and cultured overnight at 120 rpm shaking. The bacterial suspension was then centrifuged at 12,000 × *g* for 10 min to obtain precipitates, which were washed three times with 0.85 % NaCl. The cell precipitates were resuspended to a concentration of 10^9^ CFU/mL using 0.85 % NaCl and added to a beaker containing carrot cubes at a ratio of 9:1 (v/w). After stirring for 10 min, the inoculated sample was transferred to a biological safety cabinet for air-drying.

### 2.3 US-MHAsA treatment

MHAsA (1 % AsA solution at 50 °C) [8] was added to a beaker containing 30 g of carrot cubes at a ratio of 5:1 (v/w) and then placed in a US washer for 5 min. The frequency and power of the washer were 28 kHz and 300 W [17], respectively. The processed sample was dewatered using a manual salad spinner and then packaged in preservation boxes (133 × 133 × 21 mm) with a sample weight of 150 g/box [8]. The boxes were then sealed with plastic wrap and stored at 4 °C. Samples were taken out at 0, 1, 3, and 5 days for analysis.

### 2.4 Microbiological analysis

The sample (15 g) was mixed with 0.85 % NaCl (135 mL) in a sterilized stomacher bag and homogenized for 2 min to prepare the bacterial suspension. The serially diluted suspension (1 mL) was spread-plated on modified sorbitol MacConkey agar (Hopebio) and xylose-lysine-deoxycholate agar (Hopebio) for the analysis of *E. coli* O157:H7 and *S*. Typhimurium, respectively. The data was examined following a 48-h incubation period under 37 °C.

### 2.5 Cross-contamination incidence analysis

The investigation was carried out based on the research conducted by Wang et al. [18]. The concentrations of the two pathogens were modified to 10^5^–10^6^ CFU/mL using MHAsA or distilled water within 10 s. The bacterial solution was then transferred to a beaker containing uninoculated carrot cubes. The treatment procedure was described in section 2.3. The counts of the inoculated samples were assessed as outlined in section 2.4. A control sample treated with distilled water was used to calculate the incidence according to the following formula:

$$\mathrm{Cross}-contaminationincidence=\frac{\mathrm{Countsintreatmentgroup}(\log CFU/g)}{\mathrm{Countsincontrolgroup}(\log CFU/g)}$$

### 2.6 Antioxidant enzymes and reactive oxygen species (ROS) analysis

The sample was subjected to immersion in liquid nitrogen for a period of 30 s and subsequently processed for 30 s using an IKA analytical mill. The resulting powder was utilized for the analysis in 2.7, 2.8, 2.9, 2.10. The analysis of antioxidant enzymes, including SOD and CAT, was conducted using the G0101F and G0303F kits, respectively, provided by Geruisi-bio (Suzhou, China). One enzyme unit (U) of SOD was defined as the rate of inhibition of 50 % in the xanthine oxidase coupling reaction system. The decomposition of 1 μmol of H_2_O_2_ per gram of sample per minute at a temperature of 25 °C was considered equivalent to one CAT unit (U). Finally, the ROS parameters, including H_2_O_2_, O_2_^−^, and malondialdehyde (MDA), were analyzed using G0168F, G0116F, and G0109F kits, respectively, also from Geruisi-bio.

### 2.7 AsA- glutathione (GSH) cycle analysis

Enzymes, namely ascorbate peroxidase (APX) and glutathione reductase (GR), were examined using G0203F and G0209F kits, respectively, from Geruisi-bio. The content of AsA and GSH was analyzed using G0201F and G0206F kits, respectively, from the same provider. The oxidation of 1 nmol GSSG per gram tissue per minute was considered equivalent to one unit (U) of GR. Meanwhile, one unit (U) of APX activity was defined as the oxidation of 1 µmol ascorbic acid per gram tissue per minute at 25 °C.

### 2.8 Polyphenoloxidase (PPO) and peroxidase (POD) analysis

The analysis of PPO and POD activity was conducted using the G0113F and G0107F kits, respectively, provided by Geruisi-bio. A unit (U) of PPO was equivalent to a 0.01 rise in absorbance at 420 nm per gram of sample per minute, while a unit (U) of POD was represented by a 1 absorbance value increase at 470 nm per gram of tissue per minute.

### 2.9 Lignin metabolism analysis

Enzyme assays were conducted for PAL and cinnamyl alcohol dehydrogenase (CAD) using the G0114F and G1002F kits, respectively, from Geruisi-bio. Lignin content was determined using the G0708F kit from the same supplier. The determination of polyphenolic content followed a previous report [19]. PAL activity was defined as an increase in absorbance value of 0.05 at 290 nm per gram of tissue per hour at 37 °C, while CAD activity was defined as the oxidation of 1 nmol cinnamyl alcohol to form 1 nmol cinnamaldehyde per gram tissue per minute at the same temperature.

### 2.10 Statistical analysis

All experiments were independently performed three times. The data was expressed as mean value ± standard deviation. The statistical analysis was conducted using SPSS v.20 software (SPSS, Chicago, IL, USA). The Duncan module within the software was utilized to compare the mean values between each group using a multiple comparison style. A *p*-value of less than 0.05 was considered as significant difference.

## 3 Results

### 3.1 Effects of US-MHAsA on antioxidant enzymes and ROS

**Fig. 1:**
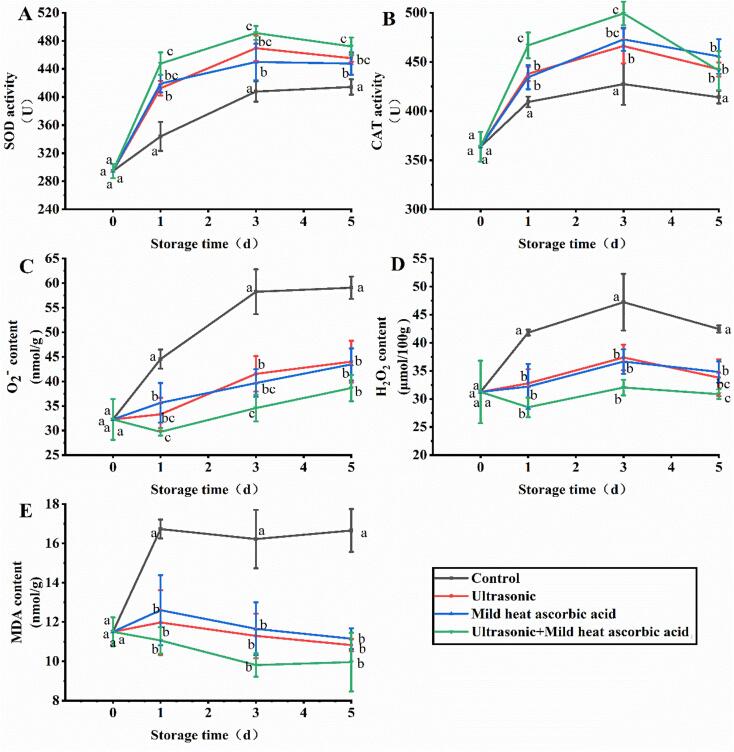
Changes of antioxidant enzymes and ROS during storage. A, B, C, D, and E indicate SOD, CAT, O_2_^−^, H_2_O_2_, and MDA, respectively. Different lowercase letters at the same day indicate significant difference (*P* < 0.05).

The results revealed that the US-MHAsA group exhibited the highest SOD activity, which was 1.07-, 1.09-, and 1.05-fold of MHAsA at days 1, 3, and 5, respectively (Fig. 1A). The SOD activity observed in the US group was comparable to that of MHAsA. All three treatment groups, including US-MHAsA, US, and MHAsA, displayed significantly higher SOD activity than the control group during storage (days 1–5). The CAT activity in the control group was 363.6–427.4 U during storage, which was significantly lower than the activity levels observed in the three treatment groups (Fig. 1B). At days 1 and 3, the CAT activity in the US-MHAsA group was significantly higher than that of the US group. The highest levels of H_2_O_2_ and O_2_^−^ were observed in the control group during storage (Fig. 1C and 1D), which were in contrast to the results observed for SOD and CAT. The lowest levels of H_2_O_2_ and O_2_^−^ were achieved by US-MHAsA, with the H_2_O_2_ content at days 1, 3, and 5 accounting 68 %, 68 %, and 73 % of the control group, and the O_2_^−^ content at days 1, 3, and 5 corresponding to 67 %, 59 %, and 65 % of the control group, respectively. The H_2_O_2_ and O_2_^−^ content in the US group was comparable to that of MHAsA, and both single treatment groups were significantly lower than the control group. The MDA levels in the three treatment groups were all significantly lower than those of the control group (Fig. 1E). The lowest MDA value (11.5–16.7 nmol/g) was observed in the US-MHAsA group during days 1–5, and the values at days 1, 3, and 5 were 66 %, 60 %, and 60 % of the control group value, respectively.

### 3.2 Effects of US-MHAsA on AsA-GSH cycle

**Fig. 2:**
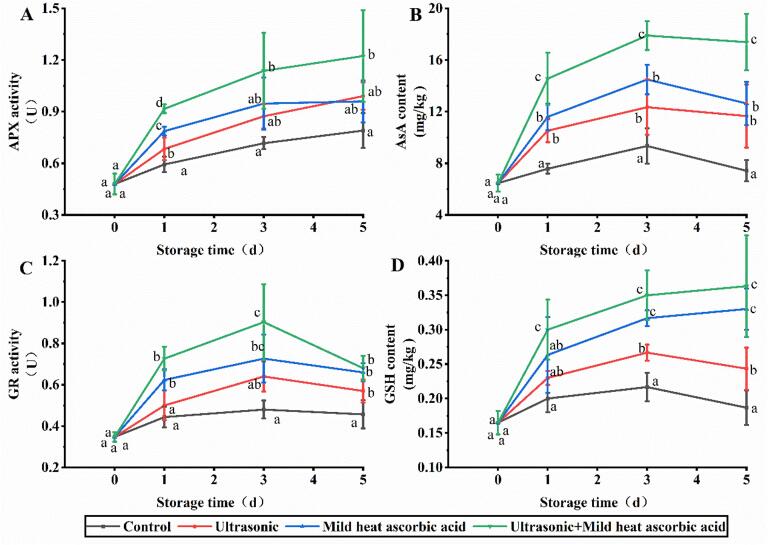
Changes of key enzymes and compounds in GSH-AsA cycle. A, B, C, and D indicate APX, AsA, GR, and GSH, respectively. Different lowercase letters at the same day indicate significant difference (*P* < 0.05).

The APX activity in the control group was 0.48–0.79 U during storage, as shown in Fig. 2A. The US-MHAsA group exhibited the highest APX activity, which was 1.54-, 1.59-, and 1.55-fold of control group at days 1, 3, and 5, respectively (Fig. 2B). Correspondingly, the US-MHAsA group lead the highest AsA content, which was significantly greater than that of the control, MHAsA, and US groups (Fig. 2B). The GR activity in the US-MHAsA group during storage was 0.68–0.90 U, and its value at days 1 and 3 was 1.45- and 1.41-fold of the control group, respectively (Fig. 2C). The GSH content in the US-MHAsA group was 0.30–0.36 mg/kg during storage, which was significantly higher than that of the US group (Fig. 2D). As shown in Fig. 2D, there was a significant difference between control and the two single treatments at days 3 and 5.

### 3.3 Effects of US-MHAsA on lignin metabolism

**Fig. 3:**
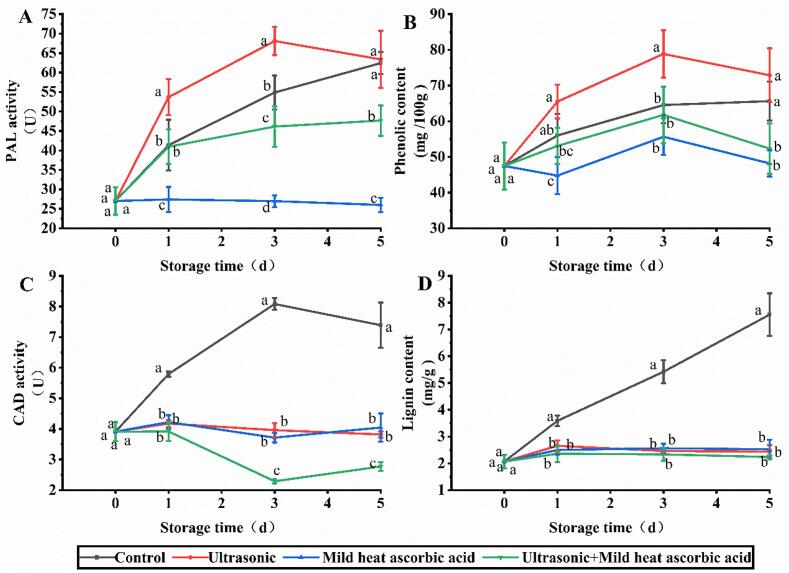
Changes of key enzymes and compounds associated with lignin metabolism. A, B, C, and D indicate PAL, phenolic, CAD, and lignin, respectively. Different lowercase letters at the same day indicate significant difference (*P* < 0.05).

The improvement in PAL activity was remarkable after treatment with US, as evidenced by the values of 53.68 and 68.11 U at day 1 and 3, respectively, which were 1.30- and 1.24-fold of that in control (Fig. 3A). From day 3 to 5, the PAL activity in the US-MHAsA group was significantly lower than that of the control. For MHAsA, the PAL activity was 40.96–47.66 U during storage, which was significantly lower than the control and the two single treatments. The trend of phenolic content was consistent with PAL; the highest (65.48–78.86 mg/100 g) and lowest (52.33–61.77 mg/100 g) values were observed in the US and MHAsA groups, respectively (Fig. 3B). For CAD, the activity in the control was 5.79–8.08 U during storage and was significantly higher than that of the three treatment groups (Fig. 3C). The lowest CAD activity was observed in the US-MHAsA group, and the observed value at day 3 and 5 was significantly lower than that in the two single treatment groups. The lignin content in the control increased from 3.59 to 7.55 mg/g during storage (day 1–5), while the content in the three treatment groups was 2.24–2.66 mg/g (Fig. 3D). No significant difference was observed between three treatment groups during storage (Fig. 3D).

### 3.4 Effects of US-MHAsA on PPO and POD

**Fig. 4:**
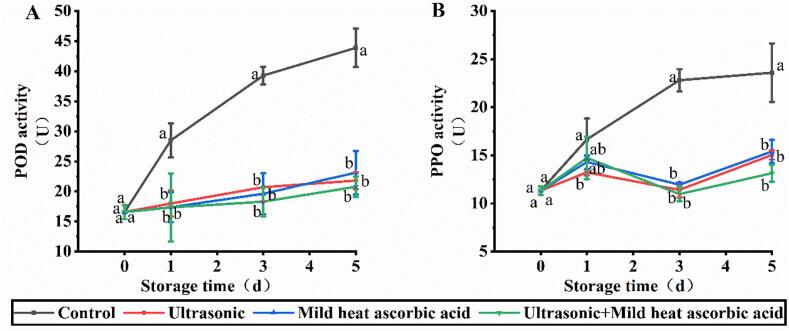
Changes of POD (A) and PPO (B) activity during storage. Different lowercase letters at the same day indicate significant difference (*P* < 0.05).

The control group exhibited an increase in POD activity from 28.49 to 43.89 U during the storage period (day 1–5), which was significantly higher than the three treatment groups (Fig. 4A). No significant difference was observed between the US-MHAsA group and the two single treatment groups. For PPO, the control group also demonstrated the highest activity during storage, and the values observed at day 3 (22.81 U) and day 5 (23.60 U) were significantly higher than the three treatment groups (Fig. 4B). The PPO inactivation capacity of the US-MHAsA group was consistent with that of the two single treatment groups, which aligns with the PPO results.

### 3.5 Inactivation efficacy of US-MHAsA

**Fig. 5:**
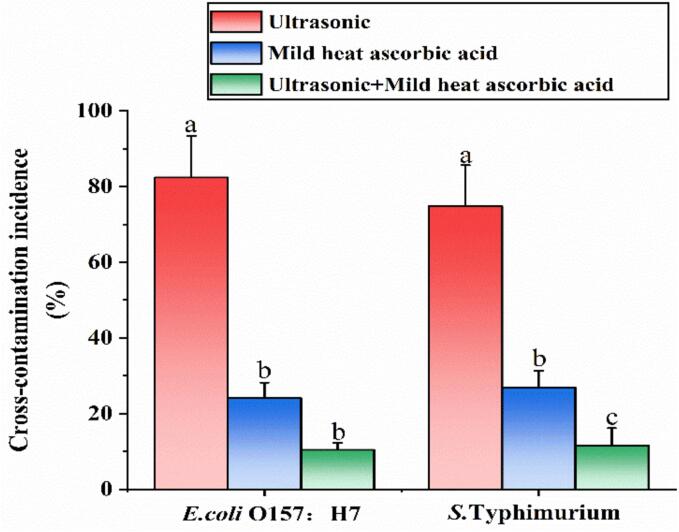
Effects of three washing methods on the cross-contamination incidence of *E. coli* O157:H7 and *S*. Typhimurium. Different lowercase letters within the same group indicate significant difference (*P* < 0.05).

After washing with US, the incidence of cross-contamination for *E. coli* O157:H7 and *S*. Typhimurium was found to be 82 % and 75 %, respectively (Fig. 5). However, the incidence for *E. coli* O157:H7 and *S*. Typhimurium was decreased to 24 % and 27 % as employing MHAsA, respectively. Notably, the lowest incidence of *E*. coli O157:H7 (10 %) and *S*. Typhimurium (12 %) was achieved by US-MHAsA.

**Fig. 6:**
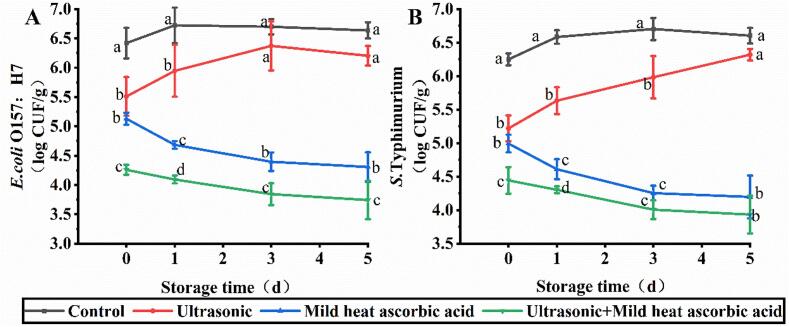
Capacity of three washing methods in inactivating *E. coli* O157:H7 (A) and *S*. Typhimurium (B). Different lowercase letters within the same day indicate significant difference (*P* < 0.05).

On day 0, the control group exhibited counts of *E. coli* O157:H7 and *S*. Typhimurium at 6.42 and 6.25 log CFU/g (Fig. 6), respectively. Following treatment with US-MHAsA, *E*. *coli* O157:H7 and *S*. Typhimurium were inactivated to 4.26 and 4.44 log CFU/g, respectively, which were lower than those in the US and MHAsA group. During days 3–5, no significant difference was observed in the counts of *E*. *coli* O157:H7 between the US and control groups. The counts of *S*. Typhimurium in the US group increased from 5.22 to 6.66 log CFU/g during storage (days 0–5), and no significant difference was observed between the control and US groups at day 5. In contrast, the counts of both pathogens decreased during storage following treatment with MHAsA and US-MHAsA. The lowest counts of *E. coli* O157:H7 (3.74–4.26 log CFU/g) and *S*. Typhimurium (3.93–4.44 log CFU/g) were observed in the US-MHAsA group.

## 4 Discussion

The accumulation of ROS in fresh-cut produce can lead to oxidative stress and initiate oxidation damage to lipids, proteins, and nucleic acids, resulting in browning, softening, vitamin degradation, and aging [20]. MDA, a lipid peroxidation product, is an important indicator for evaluating the extent of oxidative damage in fresh produce and is positively correlated with ROS content [21], [22]. SOD and CAT are important antioxidant enzymes in fruits and vegetables. SOD converts O_2_^−^ to H_2_O_2_, which is then catalyzed by CAT to produce water and oxygen [23]. As an exogenous stimulus, US can induce the expression of SOD and CAT in response to stress [24]. Similarly, MHAsA has also been reported to induce the activities of SOD and CAT in fresh-cut carrots [8]. When US and MHAsA were combined, a higher expression level of CAT and SOD was induced compared to single treatments. GSH and AsA can scavenge ROS. APX is responsible for catalyzing AsA to scavenge ROS, with the final product DHA being further reduced to AsA by GSH [25]. GSH can also scavenge ROS to form GSSG, and GR is responsible for reducing GSSG to GSH [26]. This process is known as the GSH-AsA cycle. Therefore, increasing the activity of APX and GR can accelerate the GSH-AsA cycle to scavenge ROS. A study from Wang et al [8] suggest that the activity of APX was induced by MHAsA. In previous studies, single treatments were commonly used to increase the activity of SOD and CAT and accelerate the GSH-AsA cycle [27], [28], [29]. However, in this study, a combination treatment was used and found to accelerate the AsA-GSH cycle and increase the activity of SOD and CAT compared to single treatments, resulting in the lowest ROS and MDA content.

Lignin, a polymer present in the cell walls of plants, provides structural rigidity and enhances resistance against stress [30]. However, mechanical damage caused by cutting results in the synthesis of lignin on the cut surface, which leads to quality loss. PAL is the first enzyme in the lignin synthesis pathway, catalyzing the production of phenolics from L-phenylalanine [30]. CAD is the final enzyme in the pathway, catalyzing the synthesis of lignin monomers [31]. Research has shown that US can cause compression and expansion of plant cells, and this mechanical and cavitation effect was responsible for the increased activity of PAL [32]. In this study, we also observed that US could induce PAL activity. However, MHAsA was reported to inactivate PAL [8]. This finding can explain why the activity of PAL in the US-MHAsA group was intermediate between the two single treatments. In the present study, although the inactivation capacity of US-MHAsA against CAD was more effective than the two single treatments during day 3–5, similar results were not observed for lignin content. The content of lignin increased in all groups from day 0–1, and the inhibition effect against CAD after day 1 could inhibit further synthesis of lignin rather than decreasing the content of lignin. This can explain why the lignin content in the US-MHAsA group was not significantly lower than the two single treatments.

The accelerated oxidation of phenolics on the cut surface due to increased activity of PPO and POD can lead to browning, which negatively affect the appearance, flavor, and consumer acceptance [29]. Previous studies have indicated that AsA, US, and MH are effective in inactivating PPO and POD when utilized individually [33], [34], [35]. However, MH was reported to inactivate PPO by 10 % at 40 and 50 °C for 120 min [36]. As such, the combination of US and MHAsA may not further inactivated PPO and POD in the present study, possibly due to the use of 50 °C AsA.

The recycling of washing water is a crucial aspect in the minimal processing industry. When contaminated produce is washed, pathogens can enter the water and cross-contaminate uncontaminated produce [37]. Therefore, it is essential to reduce the incidence of pathogen cross-contamination to ensure the safety of fresh-cut produce. The cross-contamination incidence of *E. coli* O157:H7 and *S.* Typhimurium exceeds 80 % as employing US alone [18]. Moreover, the inactivation efficacy of US in inactivating pathogens on produce is limited [38]. Chemical disinfectants are commonly used in combination with US to reduce the incidence to less than 30 % [16], [18], but they cannot simultaneously inactivate pathogens and improve the quality properties of fresh-cut produce. In the present study, we used MHAsA in combination with US and found that the cross-contamination incidence could be reduced to 10–12 % and the quality properties of the fresh-cut carrots were improved.

The application of US has been demonstrated to inactivate bacteria by inducing cavitation and generating localized heating, which can result in cell membrane damage [38]. MHAsA exhibited an inactivation capacity by damaging the cell membrane and disrupting intracellular processes, such as DNA replication and energy metabolism [8]. Compared to pathogens treated with US and MHAsA, those treated with US-MHAsA may experience more severe damage to their cell membranes and intracellular components, resulting in a higher inactivation efficacy and a lower incidence of cross-contamination. Coating is a promising preservation method that can continuously control the growth of pathogens on produce during storage [39]. After washing with chemical disinfectants, rinsing is required to remove any residue on produce surface [40], but this step is not necessary after washing with AsA. Additionally, US was effective during washing. Consequently, the number of pathogens decreased in the AsA and US-MHAsA groups, while the number of pathogens increased in the US group. Similarly, after washing cherry tomatoes with AsA and storing them for 5 days, the counts of *E. coli* O157:H7 and *S*. Typhimurium were significantly lower than the samples washed without AsA [19].

## 5 Conclusion

A novel low-cost and safe method called MHAsA was combined with US to enhance the quality and microbial safety properties of fresh-cut carrots. The study found that US-MHAsA induced more intense antioxidant enzymes (SOD and CAT) activities and accelerated GSH-ASA cycles, resulting in the lowest ROS (H_2_O_2_ and O_2_^−^) and MDA contents compared to US and MHAsA. Additionally, US induced the highest PAL activity and phenolic content. The inactivation efficacy of US-MHAsA against CAD was higher than that of US and MHASA, while the lignin content was insignificantly different between US-MHASA, US, and MHASA. The inactivation capacity of US-MHAsA against PPO and POD was consistent with US and MHASA. Furthermore, the disinfection efficacy of US-MHAsA was stronger than US and MHAsA as analyzing pathogens (*E. coli* O157:H7 and *S*. Typhimurium) cross-contamination incidence and present counts on the surface of carrots. However, the inactivation mechanism of US-MHAsA against the two pathogens was not studied in this research, and molecular-level investigations will be conducted in future studies.

## CRediT authorship contribution statement

**Jiayi Wang:** Writing – review & editing, Writing – original draft, Supervision, Investigation, Funding acquisition, Conceptualization. **Ning Zhou:** Writing – review & editing, Data curation. **Sen Ma:** Writing – review & editing. **Xiaofei Yang:** Writing – review & editing. **Jun Xing:** Writing – review & editing.

## Declaration of competing interest

The authors declare that they have no known competing financial interests or personal relationships that could have appeared to influence the work reported in this paper.
